# Cardiovascular MRI evidence of reduced systolic function and reduced LV mass in rheumatoid arthritis: impact of disease phenotype

**DOI:** 10.1007/s10554-019-01714-6

**Published:** 2020-02-08

**Authors:** L. A. Bissell, B. Erhayiem, E. M. A. Hensor, G. Fent, A. Burska, A. K. McDiarmid, P. P. Swoboda, H. Donica, S. Plein, M. H. Buch, J. P. Greenwood, J. Andrews

**Affiliations:** 1 Leeds Institute of Rheumatic and Musculoskeletal Medicine, University of Leeds, Leeds, UK; 2 NIHR Leeds Biomedical Research Centre, Leeds Teaching Hospitals NHS Trust, Leeds, UK; 3 Multidisciplinary Cardiovascular Research Centre & The Division of Biomedical Imaging, Leeds Institute of Cardiovascular and Metabolic Medicine, University of Leeds, Leeds, UK; 4 Medical University of Lublin, Lublin, Poland

**Keywords:** Rheumatoid arthritis, Cardiovascular disease, Cardiovascular MRI

## Abstract

The accelerated risk of cardiovascular disease (CVD) in Rheumatoid Arthritis (RA) requires further study of the underlying pathophysiology and determination of the at-risk RA phenotype. Our objectives were to describe the cardiac structure and function and arterial stiffness, and association with disease phenotype in patients with established) RA, in comparison to healthy controls, as measured by cardiovascular magnetic resonance imaging (CMR). 76 patients with established RA and no history of CVD/diabetes mellitus were assessed for RA and cardiovascular profile and underwent a non-contrast 3T-CMR, and compared to 26 healthy controls. A univariable analysis and multivariable linear regression model determined associations between baseline variables and CMR-measures. Ten-year cardiovascular risk scores were increased in RA compared with controls. Adjusting for age, sex and traditional cardiovascular risk factors, patients with RA had reduced left ventricular ejection fraction (mean difference − 2.86% (− 5.17, − 0.55) p = 0.016), reduced absolute values of mid systolic strain rate (p < 0.001) and lower late/active diastolic strain rate (p < 0.001) compared to controls. There was evidence of reduced LV mass index (LVMI) (− 4.56 g/m^2^ (− 8.92, − 0.20), p = 0.041). CMR-measures predominantly associated with traditional cardiovascular risk factors; male sex and systolic blood pressure independently with increasing LVMI. Patients with established RA and no history of CVD have evidence of reduced LV systolic function and LVMI after adjustment for traditional cardiovascular risk factors; the latter suggesting cardiac pathology other than atherosclerosis in RA. Traditional cardiovascular risk factors, rather than RA disease phenotype, appear to be key determinants of subclinical CVD in RA potentially warranting more effective cardiovascular risk reduction programs.

## Introduction

Rheumatoid arthritis (RA) is associated with an accelerated risk of cardiovascular disease (CVD), with both traditional cardiovascular risk factors and systemic inflammation playing a role [1]. To improve on the European league against rheumatism (EULAR) recommendations for reducing CVD in RA [2], there is a need for greater understanding of the underlying pathophysiology and determination of the RA phenotype most at risk of CVD.

Cardiovascular magnetic resonance (CMR) imaging provides highly reproducible quantitative assessment of the heart and cardiovascular system, with high diagnostic accuracy for ischaemic heart disease (IHD) [3]. CMR is increasingly used as a research tool in patients with RA, detecting the presence of subclinical disease in populations free of clinical CVD [4–8]. The largest CMR study in RA (n = 75, some with diabetes) reported a 4.4% reduction in LV ejection fraction (LVEF), but interestingly also a 15% reduction in LV mass index (LVMI) in those with RA [9]. A preliminary CMR report of 66 patients with treatment-naive early RA also demonstrated a reduced LVMI [10]. Both of these CMR studies stand in contrast to echocardiographic data [11] and smaller CMR studies in RA [8, 12] reporting no difference or increase in LV mass. This is of pathophysiological interest given the occurrence of heart failure in RA is not fully attributable to traditional cardiovascular risk factors or the presence of IHD [13].

This exploratory study describes cardiac structure and function and arterial stiffness in patients with established RA free of known CVD and diabetes mellitus, in comparison to healthy controls, as measured by CMR. The study also describes the association of any cardiovascular abnormalities with RA disease phenotype, to provide insight into the patients most at risk of CVD.

## Methods

Consecutive patients with RA attending rheumatology clinics between January 2011 and September 2014 at the Leeds Teaching Hospitals NHS Trust were considered for this study. Patients were eligible if between 18 and 80 years old, met 1987 ACR criteria [14], had disease for 5 years or more and no history of CVD or diabetes mellitus. Healthy controls, with no history of RA or osteoarthritis affecting their mobility, were mainly identified by asking patients with RA to ‘bring a friend’. This study had full ethical approval; REC 09/H1307/98 and REC 10/H1307/103, NRES Leeds West ethics committee. Following written informed consent obtained according to the Declaration of Helsinki, study participants were invited to undergo a cardiovascular clinical assessment, fasting blood collection and CMR.

### Clinical assessment

A clinical evaluation recorded demographic data, traditional cardiovascular risk factors and for patients with RA, disease phenotype including 3-variable DAS28-C-reactive protein (CRP) [15]) and Health Assessment Questionnaire-Disease Index [16]. Fasting lipid profile and glucose were measured, and rheumatoid factor (RF), anti-cyclic citrullinated peptide antibody (ACPA), CRP, erythrocyte sedimentation rate in those with RA. Additional blood samples were processed and stored (some at − 30 °C and some at − 80 °C) for later analysis at the Department of Biochemical Diagnostics, Medical University of Lublin, Poland; glucose (using biochemical analyser Cobas INTEGRA 400); N-terminal pro-brain natriuretic peptide (NT-proBNP) (using Cobas 6000 (immunochemistry module Cobas e601)) and insulin (using COBAS e 411 (Roche Diagnostics GmbH, Mannheim, Germany) and appropriate Roche Diagnostics assays). Homeostasis model assessment-estimated insulin resistance (HOMA-IR) was then calculated (fasting insulin (μU/ml) × fasting glucose (mg/dl)/405) [17].

### CMR imaging

**Fig. 1:**
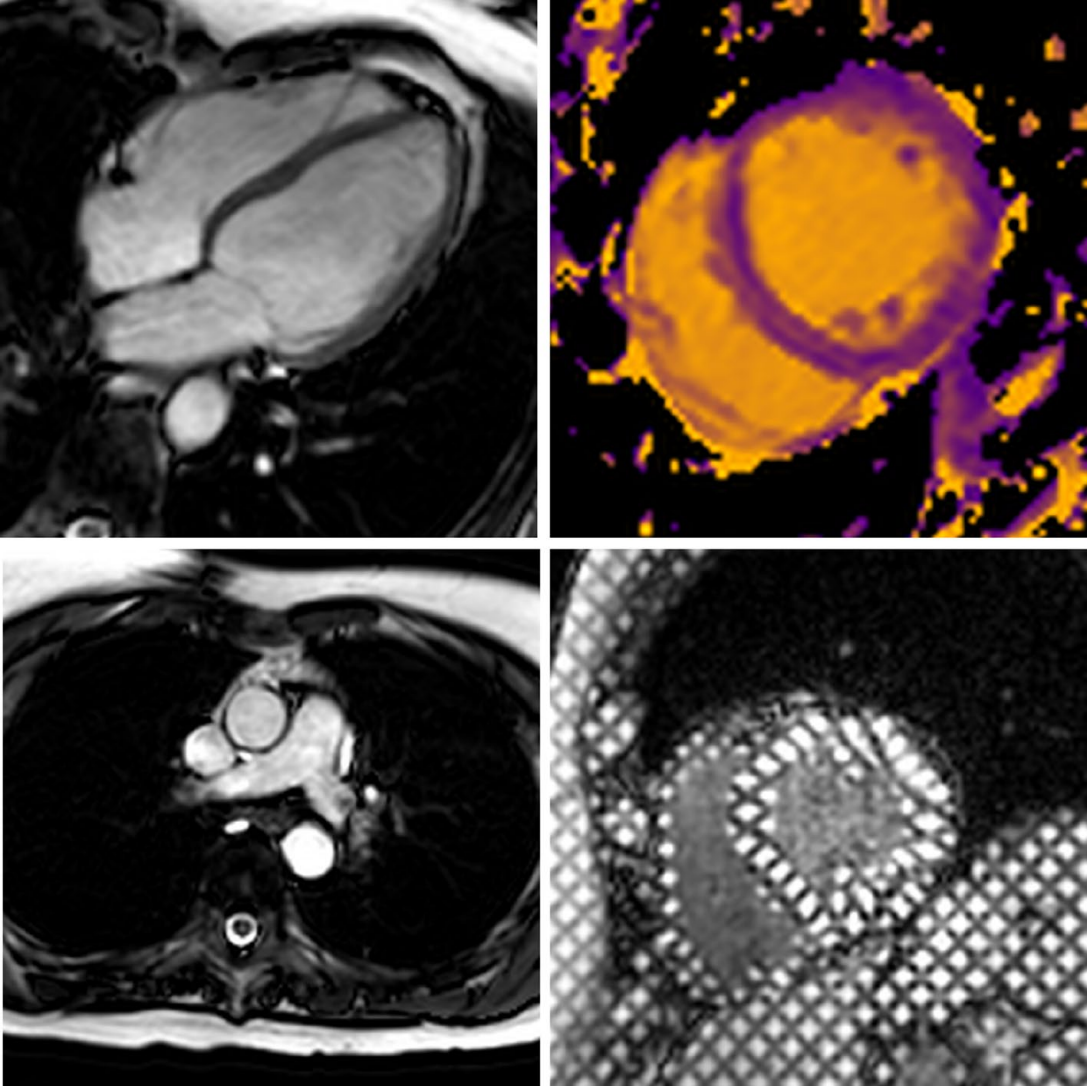
Typical CMR images from this study including SSFP cine imaging planned in the four chamber view (upper left), high temporal resolution cine imaging for aortic distensibility (lower left), native T1 mapping at mid ventricular level (upper right) and tagged cine imaging for strain analysis (lower right)

The CMR study was performed on a 3T Philips Achieva system TX equipped with a 32-channel coil. Low resolution survey, reference scans and localisers determined the cardiac short axis, vertical long axis and horizontal long axis with cine imaging (balanced steady state free precession (SSFP) acquisition, see Fig. 1). LV dimensions and function were obtained from cines covering the entire heart in the LV short axis [18, 19] (balanced SSFP, multiphase, contiguous slices, voxel size 1.2 × 1.2 × 10 mm^3^, 50 cardiac phases).

For aortic distensibility, high temporal resolution sagital-oblique and transverse cines were acquired to measure the diameter and area of the ascending aorta, descending aorta and aortic arch at the level of the main pulmonary artery [20]. Blood pressure was recorded immediately prior to image acquisition. Aortic pulse wave velocity (PWV) was assessed using identical geometry planning with retrospectively gated, through-plane, phase-contrast velocity encoded images (breath-hold, single slice, 10 mm thick, 50 phases, VENC 200 cm/s).

Tissue tagging for strain analysis and diastology were generated from the basal, mid and apical LV using the 3-of-5′ approach [21] (spatial modulation of magnetization pulse sequence, spatial resolution 1.51 × 1.57 × 10mm3, tag separation 7 mm, ≥ 18 phases, typical TR/TE 5.8/3.5 ms, flip angle 10°).

Native T1 mapping was acquired with a single breath-hold mid-slice in the LV short axis using an electrocardiogram-triggered modified Look-Locker inversion method to acquire 11 images (3-3-5 acquisition with 3 × R–R interval recovery epochs, voxel size 1.7 × 2.14 × 10 mm^3^ Trigger delay at end-diastole, flip angle 35o, FOV 320–420 mm) [22, 23].

### Data analysis

Image analysis was performed off-line blinded to patient characteristics using CMR42 (CVI42 v4.1.3, Circle Cardiovascular Imaging Inc., Calgary, Canada) and in accordance to recognised reporting standards [24]. LV dimensions and function (excluding papillary muscles) were calculated using standard criteria to delineate cardiac borders [24].

Aortic cross-sectional measurements were made by manual planimetry of the endovascular-blood pool interface, at maximal and minimal distension of the aorta. Aortic distensibility (strain/(pulse pressure (mmHg)) × 1000) [25], and aortic PWV (distance between area of ascending aorta and area of descending aorta/transit time for wave to cover distance) [25, 26] were calculated. Analysis was performed using in-house software (PMI 0.4) based on IDL 6.4 (ITT Visual Information Systems, Boulder, CO, USA) [27].

Strain analysis and LV torsion from the tagging series was derived using the open source software Osirix inTag (http://www.osrix-viewer.com). LV twist was calculated by subtracting the basal from apical rotation [28]. LV torsion takes the heart radius and length into account, describing it as the circumferential-longitudinal shear angle making the measurement comparable between hearts of different sizes and is related to myocardial fibre orientation [29]; calculated by peak twist × (apical radius + basal radius))/(2 × apex to base length).

Native myocardial T1 was measured from a desired region of interest (ROI) in the mid-ventricular wall [30]. The ROI was manually motion corrected as required from source images and care taken to avoid partial-volume effects from neighbouring tissue or blood pool.

### Statistical analysis

The statistical packages SPSS (IBM SPSS Statistics 22) and Stata/IC 13.1 were used. Following a descriptive analysis, independent t-tests determined unadjusted differences between patients with RA and controls. Linear regression determined differences when adjusted for age, sex and cardiovascular risk factors [defined as hypertension (either history of or on anti-hypertensive agent), dyslipidaemia (either history of or on lipid-lowering medication or total cholesterol/high-density lipoprotein cholesterol (TC/HDL-C) ratio greater than 6) and ever smoked]. Non-normally distributed variables (HOMA-IR and NT-proBNP) were log-transformed prior to analysis. Ordinal JBS2 scores were compared using Mann–Whitney U test. Using Holm’s method to correct for multiple comparisons [31], the threshold for statistical significance at the 5% level was set to p < 0.016.

Within the RA group, Pearson’s/Spearman’s correlation/univariable analyses were used to determine associations between baseline variables and CMR-measures, using log-transformed values when appropriate. Any variables considered to be associated with CMR-measures in the literature or strongly correlating by univariate analysis (coefficient greater than 0.3) were entered into a multivariable linear regression model.

In the event of missing serology the most recent value preceding the visit was carried forward into the data, excluding CRP due to its capacity to vary or for lipid/glucose profile, as a fasting state could not be verified.

## Results

Ninety-five consecutive patents with RA were recruited; 76 of these underwent CMR. Thirty healthy controls were recruited and 26 of these underwent CMR. Reasons for not having CMR performed included claustrophobia (RA n = 6), difficulty in contacting patient for CMR (RA n = 7, controls n = 3) or patient changing their mind (RA n = 3, controls n = 1), non-MR compatible implant (RA n = 1), patient too large for scanner (RA n = 1) and incomplete study data retrieved (n = 1).

### Study participant characteristics

Of those who underwent a CMR scan, Tables 1 and 2 outline the demographic, cardiovascular risk profile and RA disease-specific features. The mean age (standard deviation, SD) of patients with RA was 60 (9.2) years, 74% were female and 95% white. Median (interquartile range, IQR) disease duration was 16.5 (10.7, 25.7) years, 90% were seropositive for RF or ACPA and 78% had erosive disease. Patients overall were in remission; median (IQR) 3variable-DAS28 2.39 (1.15, 3.36). A significant proportion had cardiovascular risk factors, including 33% with hypertension. The control group were younger (mean (SD) age 51.8 (11.8) years) with fewer (54%) females and fewer cardiovascular risk factors.

**Table 1:** Study participant characteristics

Variable	Expressed as	RA patients n = 76	Controls n = 26
Demographics			
Age (years)	Mean, SD	60 (9.2) (range 31–78)	52.2 (11.4) (range 35–80)
Female	n %	56 (73.7)	14 (53.8)
White	n %	72 (94.7)	23/24 (95.8)
CV risk profile			
PMH hypertension	n %	25 (32.9)	2/23 (8.7)
PMH Hypercholesterolaemia	n %	19 (25)	1/23 (4.3)
Smoking status			
Never	n %	35 (46.1)	12/23 (52.2)
Ex		31 (40.8)	9/23 (39.1)
Current		10 (13.2)	2/23 (8.7)
Alcohol intake (units/week)	Median (IQR)	2 (2, 8)	2 (2, 8)
FHx premature CVD*	n %	20 (26.3)	4/22 (18.2)
Five or more fruit/vegetables daily intake (days/week)	Median (IQR)	5 (4, 7)	5 (4, 7)
Moderate exercise (mins/week)	Median (IQR)	37.5 (0, 142.5)	60 (0, 255)
Number of current anti-hypertensives	n %	10 (13.2)on 1 drug	1/23 (4.3) on 2 drugs
		8 (10.5) on 2 drugs	
		2 (2.6) on 3 drugs	
Current use of statin	n %	12 (15.8)	1/23 (4.3)
BMI	Mean, SD	26.1 (3.5)	25.0 (3.4)
Waist/hip ratio	Mean, SD	0.84 (0.08) (n = 74)	0.82 (0.09) (n = 23)
Systolic BP (mmHg)	Mean, SD	135 (20)	127 (16) (n = 25)
Diastolic BP (mmHg)	Mean, SD	80 (12)	72 (10) (n = 25)
Fasting blood collection			
Fasting TC/HDL-C ratio	Mean, SD	3.4 (1.0) (n = 73)	3.3 (1.0) (n = 21)
Fasting total cholesterol, mmol/L	Mean, SD	5.3 (1.1) (n = 75)	5.1 (0.9) (n = 21)
Fasting HDL-C, mmol/L	Mean, SD	1.7 (0.4) (n = 73)	1.6 (0.4) (n = 21)
Fasting LDL-C, mmol/L	Mean, SD	3.1 (0.9) (n = 73)	3.0 (0.9) (n = 21)
HOMA-IR	Geometric mean	1.10 (n = 71)	1.20 (n = 22)
NT-proBNP, pg/ml	Geometric mean	57.64 (n = 71)	42.17 (n = 22)

*BMI* body mass index, *BP* blood pressure, *CVD* cardiovascular disease, *FHx* family history of, *HOMA*-*IR* homeostasis model of assessment of insulin resistance, *PMH* past medical history of, *NT*-*proBNP* N-terminal pro-brain natriuretic peptide, *RA* rheumatoid arthritis, *TC/HDL*-*C* total cholesterol/high-density lipoprotein cholesterol ratio

*First degree relative with history of CVD when 60 years old or younger if relative female, and 55 years old or younger if relative male

**Table 2:** Disease specific characteristics of patients with rheumatoid arthritis

RA phenotype	Data as expressed	RA patients n = 76
Disease duration (years)	Median (IQR)	16.5 (10.7, 25.7) (range 4.2, 43.4)
Early morning stiffness (mins)	Median (IQR)	10 (10, 37.5)
History of orthopaedic joint surgery	n %	21 (27.6)
Number of orthopaedic joint surgical episodes	n %	10 (13.2)—1 episode 3 (3.9)—2 episodes 6 (7.9)—3 episodes 2 (2.6)—4 episodes
Current use of oral prednisolone	n %	4 (5.3)
Current use of non-biological DMARD	n %	62 (81.6)
Number of csDMARDs currently taking	n %	48 (63.2) taking 1 8 (10.5) taking 2 8 (10.5) taking 3
Number of previously tried csDMARDs	Median (IQR)	2 (1, 3) (range 0, 7)
Current use of biological DMARD	n %	51 (67.1)
Current TNFI users		22 (27.6)
Current Rituximab users		26 (34.2)
Current Tocilizumab users		3 (3.9)
Current Abatacept users		1 (1.3)
Number of treatment cycles in current RTX users	Median (IQR)	4 (3, 5.25) (range 1, 9)
Number of previously tried biological DMARDs	Median (IQR)	0 (0, 1)
Patient general health VAS	Median (IQR)	31 (15, 52)
28-Tender joint count	Median (IQR)	2 (0, 6)
28-Swollen joint count	Median (IQR)	0 (0, 1)
HAQ-DI	Median (IQR)	1.44 (0.53, 2.00)
3 variable DAS28CRP	Median (IQR)	2.39 (1.15, 3.36)
Erosions on hands/feet radiograph	n %	57/73 (78.1)
CRP (mg/L)	Median (IQR)	< 5 (0, 7.8)
ESR (mm/h)	Median (IQR)	14 (6, 27)
Rheumatoid factor positive (≥ 40iu/ml)	n %	53 (69.7)
ACPA positive (≥ 10U/ml)	n %	61/75 (81.3)

*ACPA* anti-citrullinated peptide antibody, *CRP* C-reactive protein, *csDMARDs* conventional synthetic DMARDs, *DAS28CRP* 28-joint disease activity score, *DMARDs* disease-modifying anti-rheumatic drugs, *ESR* erythrocyte sedimentation rate, *HAQ*-*DI* health assessment questionnaire-disability index, *RTX* rituximab, *VAS* visual assessment score

There was little difference in lipid, glucose levels, HOMA-IR and NT-proBNP levels between the groups (see Table 1). Joint British Societies-2 (JBS2) 10-year cardiovascular risk scores in patients with RA were double that of the controls (not statistically significant); median (IQR) (8.6 (4.1, 8.6)% versus 4.2 (1.2, 10.5)% in controls (p = 0.087); a significant difference seen when following EULAR guidelines (multiply risk scores by 1.5 in patients with RA [2]); median JBS2 risk difference 5.7 (95% CI 2.7, 10.2) p = 0.003 in RA.

In patients with RA, TC/HDL-C ratio was not associated with C-reactive protein, 3-variable DAS28, ACPA or RA disease duration (data not shown); with similar findings excluding patients on a statin (n = 12). There were weak, positive associations between NT-proBNP and both age and RA disease duration (r = 0.325, p = 0.006, and r = 0.278, p = 0.019 respectively). There were also weak, positive associations between HOMA-IR and both body mass index and waist/hip circumference ratio (r = 0.240, p = 0.044 and r = 0.368, p = 0.002 respectively).

### Cardiovascular magnetic resonance imaging

#### Differences between patients and controls

The CMR results are shown in Table 3. No pericardial effusions were noted. No significant valvular pathology or cardiac masses or features of cardiomyopathy were detected, although flow imaging and post-contrast imaging were not performed. Patients with RA demonstrated a reduction in absolute values for mid systolic strain rate (mid S’) reducing further in the analysis adjusted for age, sex and cardiovascular risk factors (0.227 (0.104, 0.349), p < 0.001). They also demonstrated a reduction in LVEF (mean (SD) in RA 59.1 (4.6) vs. 59.7 (4.8) % in controls); borderline significant in the adjusted analysis (mean difference − 2.858 (− 5.167, − 0.550) %, p = 0.016). Although early diastolic strain rate was similar across the groups, active/late diastolic strain rate was reduced in those with RA in the adjusted analysis (− 0.45 (− 0.67, − 0.23), p < 0.001).

**Table 3:** Cardiovascular magnetic resonance imaging measures in study participants

Variable	RA patients n = 76	Controls n = 26	Unadjusted p value for difference	Mean difference (95% CI), p value adjusted for age and sex	Mean difference (95%), p value adjusted for age/sex/cardiovascular risk factors*
LV ejection fraction (%)	59.1 (4.6) (n = 74)	59.7 (4.8)	0.560	− 2.100 (− 4.268, 0.068), 0.057	− **2.858 (**− **5.167,** − **0.550) 0.016**
LV EDV index (ml/m^2^)	79.09 (14.59) (n = 74)	91.08 (24.32)	0.024	− 5.15 (− 12.908, 1.878) 0.142	− 4.683 (− 12.275, 2.909) 0.224
LV ESV index (ml/m^2^)	32.45 (7.60) (n = 74)	36.88 (12.00)	0.032	− 0.545 (− 4.311, 3.222) 0.775	1.187 (− 2.491, 4.865) 0.523
LV mass index (g/m^2^)	36.35 (10.52) (n = 74)	44.06 (14.49)	**0.005**	− **5.421 (**− **9.615,** − **1.227), 0.012**	− 4.558 (− 8.917, -0.199) 0.041
LV mass/EDV (g/ml)	0.46 (0.10) (n = 74)	0.48 (0.10)	0.266	− 0.035 (− 0.075, 0.005), 0.086	− 0.033 (− 0.075, 0.010) 0.129
Stroke volume index (ml/m^2^)	46.29 (7.26) (n = 74)	53.47 (11.20)	**0.005**	− 4.273 (− 7.998, − 0.548), 0.025	− 3.551 (− 7.516, 0.413) 0.078
Peak mid systolic strain	− 0.225 (0.037) (n = 69)	− 0.244 (0.033) (n = 23)	0.032	0.020 (0.002, 0.039), 0.032	0.019 (− 0.001, 0.040), 0.066
Mid S’	− 1.205 (0.209) (n = 69)	− 1.398 (0.237) (n = 23)	**< 0.001**	**0.200 (0.089, 0.311) 0.001**	**0.227 (0.104, 0.349) < 0.001**
Mid E’	0.70 (0.21) (n = 69)	0.69 (0.29) (n = 23)	0.794	0.02 (− 0.003, 0.01), 0.791	0.04 (− 0.09, 0.17), 0.511
Mid A’	1.65 (0.36) (n = 68)	2.11 (0.50) (n = 23)	**< 0.001**	− **0.44 (**− **0.64,** − **0.45), < 0.001**	− **0.45 (**− **0.67,** − **0.23), < 0.001**
Peak twist (degrees)	10.77 (4.31) (n = 69)	12.38 (3.74) (n = 23)	0.113	− 2.026 (− 4.162, 0.110) 0.063	− 2.451 (− 4.823, − 0.080) 0.043
Torsion (degrees)	10.94 (3.86) (n = 68)	10.80 (4.41) (n = 23)	0.886	− 0.284 (− 2.331, 1.764) 0.784	− 0.889 (− 3.139, 1.360) 0.434
Native (inferoseptal) T1 = (ms)	1156.82 (53.07)	1186.00 (49.30)	0.017	− 30.09 (− 56.30, − 3.88) 0.025	− 34.78 (− 64.09, − 5.50) 0.021
Pulse wave velocity (m/sec)	7.8 (2.9) (n = 73)	7.2 (2.3)	0.339	− 0.289 (− 1.483, 0.905) 0.632	− 0.443 (− 1.639, 0.753) 0.464
Aortic distensibility (10^−3^mmHg^−1^)	2.60 (1.82) (n = 75)	3.83 (1.56) (n = 25)	**0.003**	− 0.340 (− 0.996, 0.316) 0.307	− 0.222 (− 0.936, 0.492) 0.538

Bold values represent statistical significance

Values expressed as mean (SD) unless stated otherwise

*A’* late/active diastolic strain rate, *E’* early diastolic strain rate, *EDV* end-diastolic volume, *ESV* end-systolic volume, *LV* left ventricular, *S’* peak systolic strain rate

*CV risk factors: hypertension (history/anti-hypertensive agent), dyslipidaemia (history/lipid-lowering medication/TC/HDL-C ratio > 6), ever smoked, premature CVD family history. Using Holm’s method for multiple comparisons correction, threshold for statistical significance at 5% level set to p < 0.016

LVMI was reduced in patients with RA (mean (SD) 36.35 (10.52) vs. 44.06 (14.49)g/m^2^ in controls, p = 0.005), and when adjusted for age and sex, but not after further adjustment for cardiovascular risk factors, although the difference remained substantive (mean difference (95% CI) − 4.56 (− 8.92, − 0.199) p = 0.041).

Whilst aortic distensibility was lower in RA in the unadjusted analysis, there was no substantive/statistical difference in the adjusted analysis between the groups. The remaining differences were substantive but did not meet the revised threshold for statistical significance.

#### Association with RA disease phenotype and soluble cardiovascular biomarkers

LVEF and LVMI were analysed further given the differences seen between the groups and provision of information on structure/function. A univariate analysis (Table 4) in those with RA found that male sex, systolic blood pressure (sysBP) and waist/hip circumference ratio were associated with increasing LVMI, with male sex and sysBP independently associated with LVMI in a multivariable linear regression analysis (MVA). Although no variables were associated with LVEF on univariate analysis (Table 5), male sex was associated with LVEF in a MVA. No RA-specific features were associated with CMR-outcomes.

**Table 4:** Univariable and multivariable analysis of variables associated with CMR measured LVMI

Variable	LVMI				
	Univariable analysis (number of observations = 74, unless otherwise stated)			Multivariable analysis R^2^ = 0.601, n = 68	
	Correlation coefficient	B (95% CI)	p value	B (95% CI)	p value
Age*	0.139	0.157 (− 0.106, 0.420)	0.238	0.006 (− 0.215, 0.228)	0.955
Male sex*	**0.665**	15.657 (11.50, 19.783)	**< 0.001**	**14.764 (**− **10.00, 19.528)**	**< 0.001**
Systolic blood pressure*	**0.344**	0.184 (0.066, 0.302)	**0.003**	**0.122 (0.027, 0.217)**	**0.013**
Ever smoked*	0.186	3.905 (− 0.948, 8.758)	0.113	3.759 (0.232, 7.285)	0.037
Body mass index	0.005	0.017 (− 0.738, 0.773)	0.964	–	–
Waist/hip circumference	**0.327**	43.661 (13.571, 73.750) (n = 72)	**0.005**	3.899 (− 22.997, 30.795)	0.773
TC/HDL-C*	0.059	0.608 (− 1.874, 3.091) (n = 71)	0.626	0.278 (− 1.462, 2.018)	0.750
HOMA-IR	0.137	0.642 (− 0.489, 1.773) (n = 69)†	0.261	–	–
NT-proBNP	0.062	0.009 (− 0.27, 0.045) (n = 69)	0.615	–	–
RA disease duration*	0.172	0.175 (− 0.060, 0.411)	0.142	0.046 (− 0.133, 0.224)	0.612
3 Variable DAS28	0.042	0.341 (− 1.585, 2.268)	0.725	–	–
ACPA*	− 0.156	− 4.2121 (10.294, 2.053) (n = 73)	0.187	2.014 (− 2.559, 6.587)	0.382
HAQ-DI	− 0.140	− 1.883 (− 5.097, 1.331) (n = 70)	0.247	–	–
History of joint surgery	0.112	2.587 (− 2.825, 7.998)	0.344	–	–
Current use of biological DMARD	− 0.008	− 0.169 (− 5.413, 5.075)	0.949	–	–

Bold values represent statistical significance

*ACPA* anti-citrullinated peptide antibody, *CRP* C-reactive protein, *DAS28CRP* 28-joint disease activity score, *DMARD* disease-modifying anti-rheumatic drug, *HAQ*-*DI* health assessment questionnaire-disability index, *HOMA*-*IR* homeostasis model of assessment of insulin resistance, *LVMI* LV mass index, *NT*-*proBNP* N-terminal pro-brain natriuretic peptide, *TC/HDL*-*C* total cholesterol/high-density lipoprotein cholesterol ratio

*Entered into linear regression model as associated with LV mass in the literature

†Excluding high outlier: Correlation coefficient B − 0.025 (95% CI − 2.732, 2.195) p = 0.828

**Table 5:** Univariable and multivariable analysis of variables associated with CMR measured LVEF

Variable	LVEF				
	Univariate analysis (n = 74, unless otherwise stated)			Multivariable analysis R^2^ = 0.139, n = 71	
	Correlation coefficient	B (95% CI)	p value	B (95% CI)	p value
Age*	0.152	0.075 (− 0.039, 0.190)	0.195	0.079 (− 0.041, 0.200)	0.191
Male sex*	− 0.281	− 2.897 (− 5.219, − 0.574)	0.015	− **3.246 (**− **5.629,** − **0.863)**	**0.008**
Systolic blood pressure*	0.107	0.025 (− 0.030, 0.080)	0.365	0.022 (− 0.036, 0.081)	0.452
Ever smoked*	0.034	0.314 (− 1.847, 2.474)	0.773	0.069 (− 2.048, 2.185)	0.948
Body mass index	0.092	0.129 (− 0.200, 0.458)	0.437	–	–
Waist/hip circumference	− 0.022	− 1.279 (15.390, 12.833) (n = 72)	0.857	–	–
TC/HDL-C*	0.072	0.319 (− 0.746, 1.384) (n = 71)	0.553	0.333 (− 0.698, 1.363)	0.521
HOMA-IR	− 0.008	− 0.017 (− 0.522, 0.489 (n = 69)†	0.410	–	–
NT-proBNP	0.064	0.004 (− 0.012, 0.020) (n = 69)	0.850	–	–
RA disease duration	− 0.024	− 0.011 (− 0.115, 0.094)	0.851	–	–
3 Variable DAS28	0.047	0.169 (− 0.674, 1.012)	0.690	–	–
ACPA	0.020	0.235 (− 2.523, 2.994) (n = 73)	0.866	–	–
HAQ-DI	− 0.050	− 0.293 (− 1.722, 1.136) (n = 70)	0.448	–	–
History of joint surgery	− 0.001	− 0.014 (− 2.398, 2.369)	0.990	–	–
Current use of biological-DMARD	0.128	1.250 (− 1.027, 3.527)	0.277	–	–

Bold values represent statistical significance in multivariable analysis

*ACPA* anti-citrullinated peptide antibody, *CRP* C-reactive protein, *DAS28CRP* 28 joint disease activity score, *DMARD* disease-modifying anti-rheumatic drug, *HAQ*-*DI* health assessment questionnaire-disability index, *HOMA*-*IR* homeostasis model of assessment of insulin resistance, *LVEF* left ventricular ejection fraction, *NT*-*proBNP* N-terminal pro-brain natriuretic peptide, *S’* peak systolic strain rate, *TC/HDL*-*C* total cholesterol/high-density lipoprotein cholesterol ratio

*Entered into linear regression model as associated with LVEF in the literature

†Excluding high outlier: Correlation coefficient 0.108 B 0.521 (95% CI − 0.585, 1.627) p = 0.350

## Discussion

In a population of established RA free of CVD and diabetes mellitus, combining clinical assessment with measurements of soluble biomarkers of CVD and CMR, this study reports a reduction in systolic function (LVEF, peak mid systolic strain rate), early/mid diastolic strain rate and LVMI after controlling for age, sex and traditional cardiovascular risk factors, and the association of surrogate measures of CVD with traditional cardiovascular risk factors rather than RA disease-specific features.

The only similar sized CMR study in RA (n = 75) by Giles et al. also reported a 4.4% reduction in LVEF and 15% reduction in LVMI in the adjusted mean values in patients free of known CVD compared to controls (n = 225), after adjustment for blood pressure, heart rate, HDL-C, triglycerides, habitual exercise and coronary calcium score [9]. Although age, smoking habits and statin use in their study were comparable to our study, there were fewer females (52%), fewer white (88%), greater burden of hypertension (55%) and importantly, inclusion of patients with diabetes (4%). Their patients with RA may have been subjected to a smaller burden of inflammation than in our study (shorter disease duration and fewer on biological disease modifying anti-rheumatic drugs (49%)) however they were not in remission (mean DAS28 3.51). Our study demonstrates similar results in patients free of diabetes with a reduction in the adjusted mean values in LVEF of 5.11% (intercept 55.884, B − 2.858 (95% CI − 5.167, − 0.550) p = 0.016) and LVMI of 15.5% (intercept 29.456, B − 4.558 (95% CI − 8.917, − 0.199) p = 0.041) for patients with RA, who were overall in remission, i.e. CMR abnormalities despite no active RA disease.

Other CMR studies to date reporting LVEF have been inconsistent involving smaller cohorts [12] and shorter disease duration [8]. Although Ntusi et al. reported similar LVEF, they did determine reduced peak systolic circumferential strain and reduced diastolic strain rate in those with RA [8]. Similarly, smaller CMR studies have not described reduced LV mass [8, 12], although our research group has reported preliminary findings of lower LV mass, in treatment-naive early RA patients (n = 66) [10].

Echocardiography studies have repeatedly reported increased LV mass in patients with RA [32], and often no difference in LVEF [11, 33], although reduced LVEF has been associated with active disease [33]. CMR has now demonstrated greater reproducibility over echocardiography in determination of LV systolic function [34] and greater sensitivity to detect small differences in LVEF and LV mass [35], and is now widely accepted as the reference standard [34, 36].

The reason for reduced LVMI in RA is unclear, especially given that LV mass usually increases with cardiovascular risk [36]. Indeed, the ten-year cardiovascular risk scores in this study correlated strongly with increasing LV mass (rho = 0.473 for JBS2 scores, p < 0.001). Possible causes for lower LVMI in RA patients could include physical deconditioning, myocarditis [9], microvascular dysfunction [37], or even cardiac remodelling [38] given abnormal geometry in RA has been reported previously [39]. The reduction in LVMI may also reflect the pathological processes of ‘RA-cachexia’ (loss of muscle mass with increase in fat mass) [40]. Although RA cachexia is a cytokine driven process, studies fail to demonstrate its improvement following suppression of disease activity [40].

This study also demonstrated that traditional cardiovascular risk factors associated with surrogate measures of subclinical CVD, rather than RA specific features. The European Guidelines on CVD prevention provide clear recommendations applicable to those with RA [41], with lower cardiovascular event rates associated with improvement of cardiovascular risk factors in RA [42]; however, in practice, the management of cardiovascular risk in RA is suboptimal [43]. This should therefore become a priority for the reduction of CVD in RA.

## Limitations

Although relatively large compared to previous studies, this exploratory study was not powered, however, we feel the findings are worth further consideration and validation. This cross-sectional study was also unable to quantify the burden of inflammation patients with RA were exposed to over time. The cohort had established severe disease with many requiring multiple/biological disease-modifying anti-rheumatic drugs, however, they were also in remission. In the absence of previous regular measures of disease activity/inflammation, disease duration in this cohort is the proxy measure for burden of disease. The cumulative exposure to corticosteroids (known to increase LV mass) was not quantified and it was not possible to measure the effect of DMARDs, although published data suggests DMARDs can reduce arterial stiffness and CV risk most likely through the reduction of disease activity and systemic inflammation [2].

We were surprised to see a trend for reduced native (inferoseptal) T1 in those with RA. We repeated the analysis for global T1 (average of all myocardial segments) measurements and found no such difference between the groups (1111.80 (52.35) in RA vs. 1111.47 (53.37) in controls, p = 0.978. We acknowledge the addition of post-contrast T1 measurements to derive extra-cellular volume, would have provided greater detail on myocardial tissue composition.Future research agenda.

Larger and longitudinal studies of CMR in RA are required to validate these findings, determine at which point the changes occur, and if they are amenable for improvement with the treatment of RA in the biologic era. Further work is required to understand the underlying pathophysiology, particularly of the reduced LVMI, including any relationship with RA-cachexia. There is also a need for the development of effective cardiovascular risk reduction programs. We also acknowledge defining subclinical cardiovascular disease using CMR imaging is currently difficult given the multiple domains/measurements. A future area of focus for cross-speciality collaborations such as ours should be to define how subclinical disease is reported.

## Conclusions

In summary, this CMR study has demonstrated that patients with established RA and no history of CVD have evidence of reduced LV systolic function and LVMI after adjustment for traditional cardiovascular risk factors. The reduction in LVMI suggests cardiac pathology other than atherosclerosis in RA. Traditional cardiovascular risk factors appear to be key determinants of subclinical CVD in RA potentially warranting more effective cardiovascular risk reduction programs.
